# A Genital-Sparing Robot-Assisted Radical Cystectomy With Intracorporeal Urinary Diversion: A Case Report Using the hinotori™ Surgical Robot System

**DOI:** 10.7759/cureus.86685

**Published:** 2025-06-24

**Authors:** Hisanori Taniguchi, Sho Kiyota, Junichi Ikeda, Hidefumi Kinoshita

**Affiliations:** 1 Department of Urology and Andrology, Kansai Medical University, Hirakata, JPN

**Keywords:** bladder cancer, genital-sparing cystectomy, hinotori surgical system, pelvic organ prolapse, robot-assisted radical cystectomy

## Abstract

In this report, to the best of our knowledge, we present the first case of genital-sparing robot-assisted radical cystectomy (RARC) using the hinotori™ Surgical Robot System (Medicaroid Corporation, Kobe, Japan) in a 78-year-old woman with muscle-invasive bladder cancer and a history of total hysterectomy. An anterior vaginal wall-sparing cystectomy and intracorporeal ileal conduit reconstruction were completed without complications. The postoperative course was uneventful, and pathological analysis revealed pT0N0M0. This case demonstrates the feasibility and safety of genital-sparing RARC with the hinotori system and highlights its potential role in preventing postoperative pelvic organ prolapse in selected female patients.

## Introduction

The hinotori™ Surgical Robot System (Medicaroid Corporation, Kobe, Japan) is a novel robot-assisted surgical platform developed in Japan and approved for clinical use in 2020 [1]. Its application in urologic surgery - including robot-assisted radical prostatectomy, nephrectomy, and cystectomy - has demonstrated feasibility and safety, offering advantages such as three-dimensional visualization and enhanced surgical precision [2-5]. Robot-assisted radical cystectomy (RARC) has become the standard approach for muscle-invasive bladder cancer (MIBC) due to its minimally invasive nature. However, in female patients, standard radical cystectomy often includes removal of adjacent genital organs, which may lead to complications such as postoperative pelvic organ prolapse and negatively impact quality of life [6,7]. Genital-sparing techniques have been proposed to address these issues, showing promising functional and oncological outcomes in selected patients [8,9]. While such approaches have been reported using the da Vinci Surgical System (Intuitive Surgical, Sunnyvale, CA, USA), no cases have been documented using the hinotori system. Herein, to our knowledge, we report the first case of genital-sparing RARC with intracorporeal urinary diversion (ICUD) performed using the hinotori Surgical Robot System in a woman with a prior total hysterectomy.

## Case presentation

**Figure 1:**
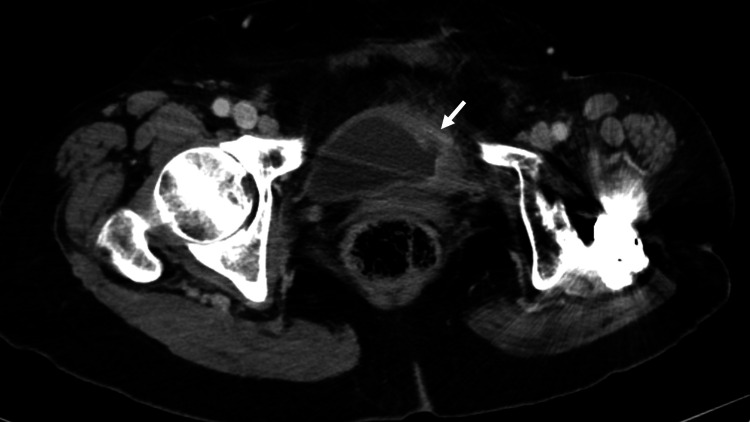
Computed tomography imaging showing an invasive bladder tumor extending from the bladder dome to the left lateral wall (arrow)

A 78-year-old woman with a history of left laparoscopic radical nephroureterectomy, performed 15 months earlier, was diagnosed with clinically invasive bladder cancer extending from the bladder dome to the left lateral wall on follow-up flexible cystoscopy. Four months post-nephroureterectomy, she underwent transurethral resection of the bladder tumor (TUR-BT), followed by intravesical Bacillus Calmette-Guérin (BCG) therapy. A subsequent repeat TUR-BT confirmed MIBC. Computed tomography (CT) revealed no lesions in the right upper urinary tract and no evidence of metastasis, with clinical staging of cT2N0M0 (Figure 1). Given the recurrence of BCG-refractory MIBC, RARC was planned.

RARC was performed using the hinotori system. The patient had previously undergone a total hysterectomy for uncontrolled postpartum hemorrhage. Considering this surgical history and the location of the tumor, an anterior vaginal wall-sparing RARC with ICUD, using an ileal conduit, was selected to minimize the risk of postoperative vaginal enterocele.

Surgical procedure

**Figure 2:**
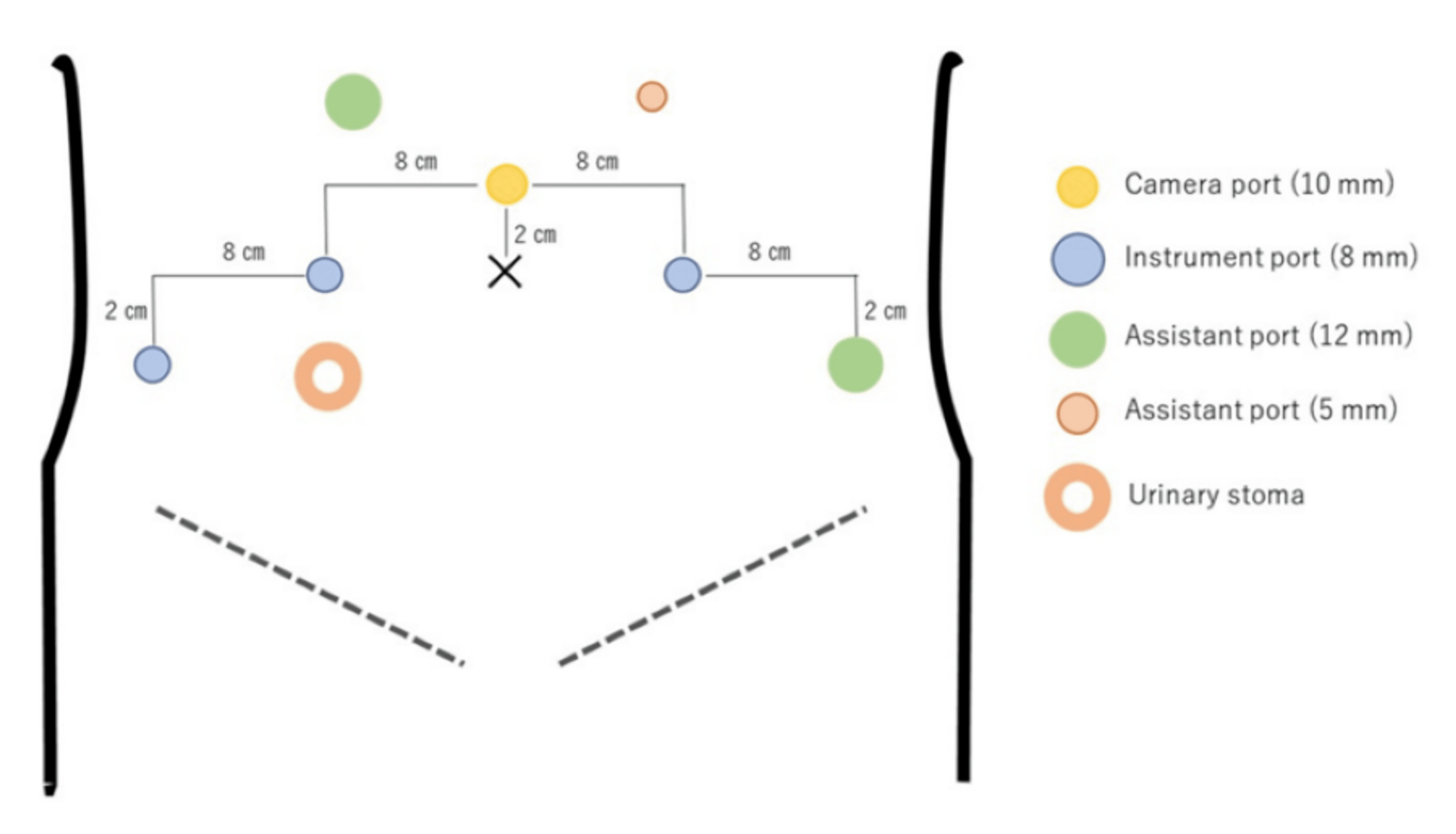
Port placement for RARC with ICUD using the hinoroti™ Surgical Robot System RARC, robot-assisted radical cystectomy; ICUD, intracorporeal urinary diversion Image Credit: Hisanori Taniguchi

After induction of general anesthesia, the patient was placed in the lithotomy position. Seven ports were established: four for the robotic arms and three for the assistant, including one with the AirSeal® System (CONMED, Utica, NY, USA) (Figure 2). A 12 mm assistant port was placed on the right side to accommodate the Signia™ Stapling System (Medtronic, Dublin, Ireland), with a 60 mm Camel reload, for the ileal conduit reconstruction.

To delineate the dissection plane between the anterior vaginal wall and the bladder, a spurtel was transvaginally inserted using the Lock Arm (Create Medic Co., Ltd., Yokohama, Japan). The patient was then adjusted to a 25° Trendelenburg position, and port placement was completed. The hinotori operation unit was positioned on the patient's right side.

**Figure 3:**
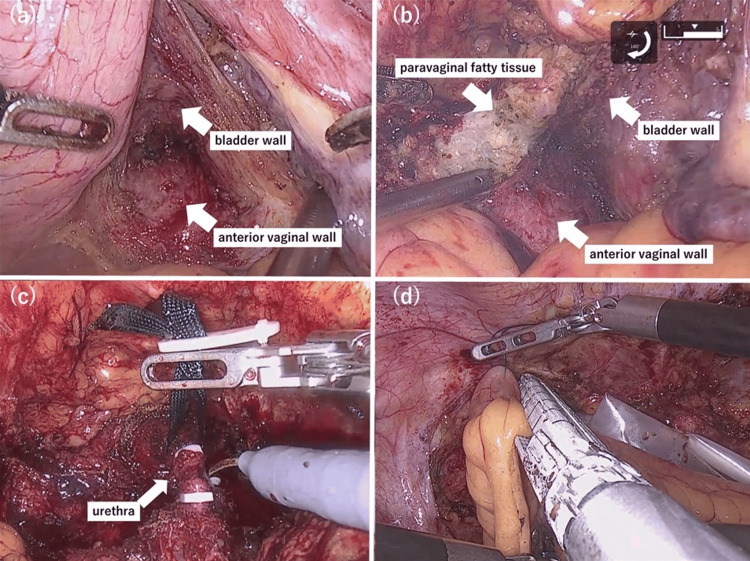
Intraoperative photographs (a) Dissection of the plane between the anterior vaginal wall and the bladder. (b) Dissection of paravaginal tissue. (c) Exposure of the urethra, followed by transection using Hem-o-Lok clips applied to the proximal and distal ends, with subsequent mobilization of the bladder. (d) Stapling of the oral end of the ileal conduit using an automatic anastomosis device inserted through the 12 mm assistant port. Croce Grasper forceps were used for bowel manipulation during urinary diversion, in place of the ProGrasp™ forceps.

A right-sided transperitoneal lymphadenectomy was performed, followed by dissection of the right ureter to the ureterovesical junction. An anterior vaginal wall-sparing radical cystectomy with urethral resection was then performed. In brief, the peritoneal attachments between the bladder and vagina were divided. Dissection of the plane between the anterior vaginal wall and the bladder was performed toward the urethra, using transvaginal insertion of the spurtel, aiding identification of the anterior vaginal wall (Figure 3a).

The retropubic space was accessed, bilateral endopelvic fasciae were incised, and the lateral vesical ligaments were divided using the LigaSure™ device (Medtronic, Dublin, Ireland). During paravaginal dissection, the presence of fatty tissue delineated the boundary of the vaginal wall, defining the dissection plane (Figure 3b). The right ureter was clipped, transected, and confirmed negative for malignancy on intraoperative frozen section. The dorsal vein complex was divided, and the anterior surface of the urethra was exposed. The urethra was mobilized dorsally using robotic forceps, followed by removal of the urethral catheter. Urethral transection was performed using Hem-o-lok XL clips (Teleflex Incorporated, Wayne, PA, USA) on the proximal and distal ends (Figure 3c). The bladder was then resected and secured in a specimen retrieval bag.

ICUD using an ileal conduit was subsequently performed. The hinotori system was temporarily undocked, and the Trendelenburg angle was adjusted to 15° before re-docking for the urinary diversion phase. Construction of the ileal conduit followed the technique described by Nakanishi et al. [10]. Resection of the ileal segment was facilitated by insertion of the Signia™ stapler through the right-sided 12 mm assistant port (Figure 3d). The right ureter was anastomosed to the ileal conduit using 4-0 polydioxanone suture, following the Wallace technique. After the procedure, the bladder specimen was retrieved through the camera port by slightly enlarging the incision site.

The total operative and console times were 446 and 328 minutes, respectively. Estimated blood loss was 367 mL, with no requirement for blood transfusion. Final pathological analysis revealed pT0N0. The postoperative course was free of complications, and the patient was discharged on postoperative day 23 without a ureteral stent and with stable renal function (Table 1).

**Table 1:** Summary of intraoperative and postoperative outcomes in the present case

Parameter	Value
Total operative time	446 minutes
Console time	328 minutes
Estimated blood loss	367 mL
Blood transfusion	None
Postoperative complications	None
Length of hospital stay	23 days
Pathological staging	pT0N0M0

## Discussion

Although definitive evidence is lacking regarding an increased incidence of postoperative vaginal enterocele following robotic surgery compared to laparoscopic or open radical procedures, previous studies have reported a risk of pelvic organ prolapse in female patients after RARC. These findings underscore the importance of including potential pelvic organ prolapse in preoperative informed consent discussions [6,7]. Preservation of the anterior vaginal wall, or pelvic floor reconstruction, might reduce the risk of such complications.

Several studies have reported on genital-sparing RARC using the da Vinci Surgical System. Roshdy et al. demonstrated favorable functional and oncological outcomes in 24 patients who underwent gynecologic-tract-sparing cystectomy for invasive bladder cancer [8], excluding those with diffuse carcinoma in situ or compromised genital tract conditions. Similarly, Moursy et al. reported successful outcomes in 18 premenopausal, sexually active women with no involvement of the bladder neck or posterior bladder wall, emphasizing the role of careful patient selection in achieving optimal results [9].

In previous reports of genital-sparing RARC performed using the da Vinci Surgical System, total operative times have ranged from approximately 360 to 540 minutes, with console times typically between 280 and 400 minutes [8,9]. In the present case, the total operative time was 446 minutes, and the console time was 328 minutes, which are within this reported range. These findings suggest that the hinotori™ system is comparable in efficiency to established robotic platforms, even in complex procedures such as genital-sparing cystectomy, and support its feasibility for broader clinical application.

Previous reports have also demonstrated the feasibility of RARC with intracorporeal ileal conduit diversion using the hinotori™ Surgical Robot System, including a 20-case series reported by Watanabe et al., which highlighted the operative safety and reproducibility of the platform [11]. However, their study did not specifically address genital preservation techniques. To our knowledge, this is the first report to focus on a genital-sparing approach using the hinotori system in a female patient. This adds a new dimension to the existing evidence by introducing a technique aimed at reducing postoperative pelvic organ prolapse in appropriately selected women.

According to the American Urological Association/American Society of Clinical Oncology/Society of Urologic Oncology guidelines, standard total cystectomy in women typically involves resection of adjacent genital organs along with the bladder [12]. In contrast, the National Comprehensive Cancer Network guidelines recommend consideration of uterus, vaginal, and ovarian preservation in appropriately selected female patients [13]. These guidelines highlight that surgical decisions regarding genital organ preservation should be individualized rather than standardized. The extent of resection should be guided by tumor location, disease extent, and patient-specific factors. A genital-sparing approach requires careful evaluation through detailed preoperative imaging and thorough intraoperative assessment.

The decision to preserve the anterior vaginal wall in this case was based on careful preoperative and intraoperative evaluations. Preoperative contrast-enhanced CT revealed that the tumor was confined to the bladder dome and left lateral wall, without evidence of extension to the bladder neck, trigone, or adjacent organs. Intraoperative findings confirmed the absence of direct invasion into the anterior vaginal wall, and no carcinoma in situ was identified on TUR specimens. Given these factors, anterior vaginal wall preservation was deemed oncologically safe in this case.

In this case, the patient had a prior total hysterectomy. Anterior vaginal wall-sparing RARC using the hinotori™ Surgical Robot System was successfully performed without complications. The patient achieved favorable oncological outcomes and did not develop postoperative vaginal enterocele. Although functional outcomes such as urinary continence and sexual function were not evaluated in this case - largely due to the patient’s advanced age and surgical history - we acknowledge this as a limitation of the current report. Future studies should incorporate systematic postoperative assessments to better evaluate the functional advantages of genital-sparing approaches and enhance their applicability in broader clinical practice. Nonetheless, to the best of our knowledge, this represents the first reported case of genital-sparing RARC performed using the hinotori™ Surgical Robot System.

## Conclusions

This report presents the case of genital-sparing RARC with ICUD performed using the hinotori™ Surgical Robot System in a female patient with prior hysterectomy. The procedure was completed safely, without perioperative complications, and achieved a favorable oncological outcome. By preserving the anterior vaginal wall, this approach may help reduce the risk of postoperative pelvic organ prolapse in appropriately selected patients. Our experience suggests that genital-sparing RARC using the hinotori system is a feasible and safe option that may contribute to better postoperative anatomical and functional outcomes in women. Further clinical accumulation is necessary to determine its broader applicability and long-term results.
